# Plasma untargeted metabolomics reveals metabolic signatures associated with epilepsy and anti-seizure medication responsiveness

**DOI:** 10.3389/fmolb.2026.1885513

**Published:** 2026-09-23

**Authors:** Zijian Li, Zhen Liang, Juan Ren, Songyan Liu

**Affiliations:** Department of Neurology, China-Japan Union Hospital of Jilin University, Changchun, China

**Keywords:** drug-resistant epilepsy, epilepsy, machine learning, metabolites, transcriptomics, untargeted metabolomics

## Abstract

**Background:**

Epilepsy is a neurological disorder with heterogeneous anti-seizure medication responses and a lack of peripheral biomarkers for diagnosis and drug resistance assessment. This study aims to explore metabolic alterations and candidate metabolite panels associated with epilepsy and anti-seizure medication responsiveness.

**Methods:**

Untargeted metabolomic analysis was performed on plasma samples from 42 patients with epilepsy (PWE), including 20 patients with drug-resistant epilepsy (DRE) and 22 patients with drug-sensitive epilepsy (DSE), as well as 20 healthy controls (HCs). Multivariate and univariate statistical analyses, pathway analysis, machine-learning-based feature selection, and logistic regression modeling were conducted, with internal validation by bootstrap resampling. Transcriptomic data were analyzed and intersected with metabolomic findings at the Kyoto Encyclopedia of Genes and Genomes (KEGG) pathway level, followed by construction of a gene–metabolite–pathway network.

**Results:**

Between the PWE and HC groups, 3,329 significantly different features were identified. KEGG enrichment analysis showed that the differential metabolites were mainly enriched in the cyclic adenosine monophosphate signaling pathway, hypoxia-inducible factor-1 signaling pathway, and dopaminergic synapse pathway, and these three pathways were also enriched in the transcriptomic dataset. Three key candidate metabolites—palmitoylcarnitine, C18 (plasm)-18:1 PC, and 1-palmitoyl-2-oleoyl-sn-glycero-3-phosphocholine—effectively discriminated PWE from HCs. Between the DRE and DSE groups, 1,074 differential ion features were identified. Six key candidate metabolites—L-kynurenine, indolelactate, glycodeoxycholic acid, N-acetylcytidine, citric acid, and 5-hydroxyindoleacetate—showed exploratory discriminatory ability between the DRE and DSE groups.

**Conclusion:**

This study revealed plasma metabolic signatures associated with epilepsy and anti-seizure medication responsiveness. The identified candidate metabolites and pathways provide preliminary metabolic evidence that requires further validation in independent cohorts and mechanistic studies.

## Graphical Abstract

**Figure:**
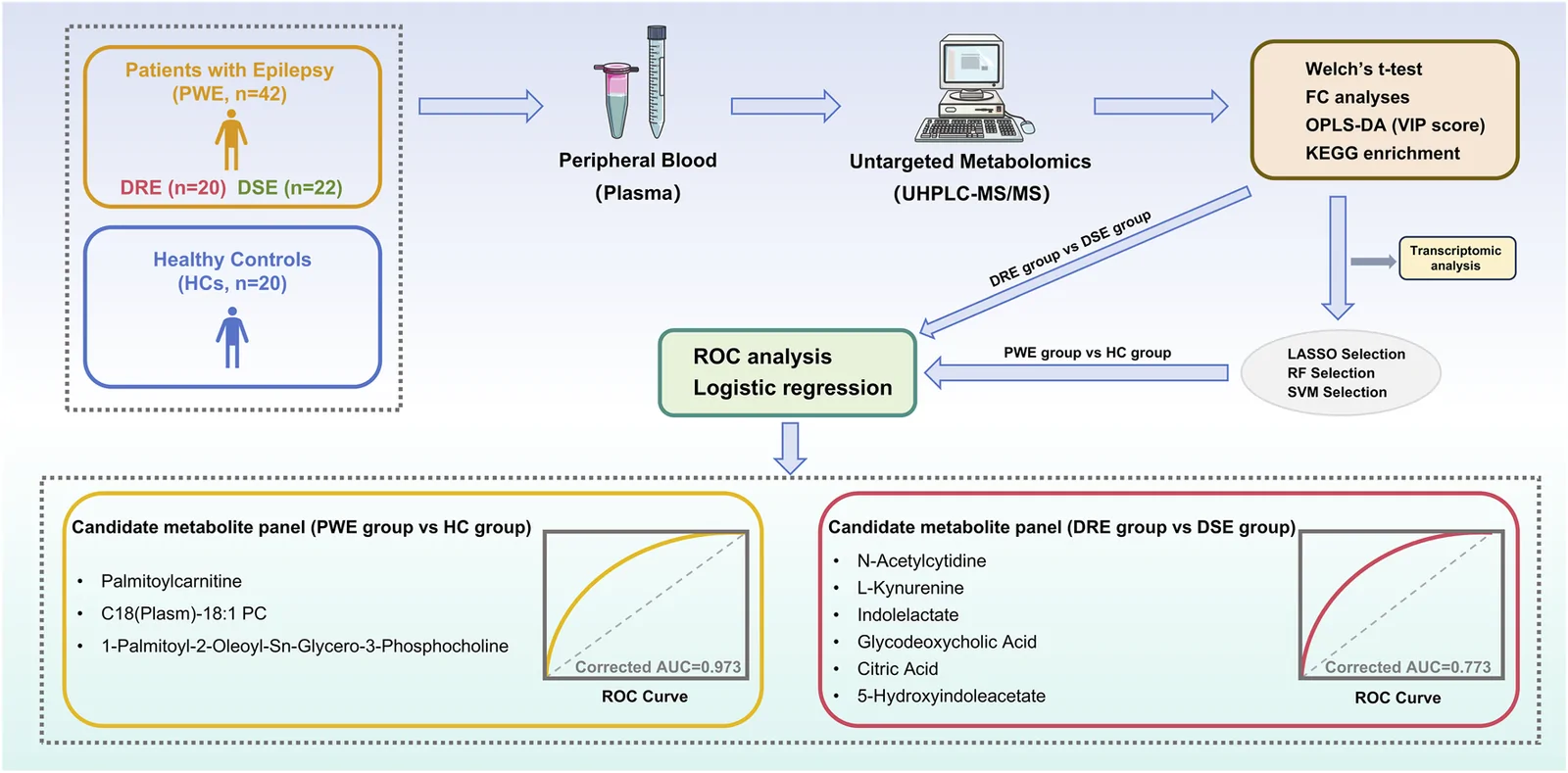
This study integrated plasma untargeted metabolomics, machine-learning-based feature selection, and transcriptomic analysis to characterize metabolic signatures associated with epilepsy and anti-seizure medication responsiveness.

## 1 Introduction

Epilepsy is a brain disorder marked by paroxysmal, transient, repetitive, and stereotypical manifestations resulting from abnormal neuronal discharge in the brain. This disorder affects approximately 70 million individuals worldwide. Despite the continuous development of anti-seizure medications (ASMs), approximately 30% of patients develop drug-resistant epilepsy (DRE) due to an incomplete understanding of its pathophysiological mechanisms. Moreover, the diagnosis of epilepsy currently relies primarily on clinical symptoms and electroencephalography with a conspicuous absence of specific biomarkers (Thijs et al., 2019). Nevertheless, accumulating evidence suggests a complex bidirectional relationship between metabolic alterations and epilepsy. Consequently, elucidating the pathophysiological mechanisms underlying epilepsy from a metabolic perspective has garnered considerable research interest and holds promise for the discovery of epilepsy biomarkers and novel therapeutic targets (Lai et al., 2022).

Metabolomics, a scientific approach that elucidates the relationships between diseases and metabolites, molecular intermediates, and metabolic products, has a vital function in investigating the pathophysiology of epilepsy and identifying potential biomarkers and therapeutic targets. Studies have shown that metabolic profiles vary across different types of epilepsy syndromes, origins of epileptic discharges, etiologies, and even among different brain regions in the same patient (Lai et al., 2022). Metabolic alterations are established causative factors of epilepsy, and epileptic seizures can themselves provoke further metabolic changes (Guo et al., 2023; Hyppönen et al., 2023; Tan et al., 2018). For example, Akiyama et al. (2020) reported reduced levels of 2-ketoglutaric acid in the cerebrospinal fluid of children with epilepsy aged 0–5 years, along with elevated levels of pyridoxamine and tyrosine. In patients with temporal lobe epilepsy, the hippocampal tissue exhibits a distinct shift in the polarity of phospholipid metabolism; positively charged phospholipids, including phosphatidylcholines and phosphatidylethanolamines, are markedly decreased, while their negatively charged counterparts, phosphatidylserines and phosphatidylglycerols, are increased. This complementary alteration in the lipid profile likely results from the dysregulation of key enzymes involved in phospholipid synthesis and degradation pathways, as well as the metabolic crosstalk and interconversion among different pathways. This disruption of hippocampal lipid homeostasis in temporal lobe epilepsy suggests reprogramming of the metabolic network (Ajith et al., 2021; Mondal et al., 2024). Additionally, the ketogenic diet, a metabolic therapeutic approach, has demonstrated efficacy in managing DRE, suggesting that DRE may arise from metabolic dysfunction (Zarnowska, 2020). Moreover, the relationship between metabolic abnormalities and neurological disorders has attracted increasing attention in recent years. For instance, metabolomic studies of cerebrospinal fluid from patients with Alzheimer’s disease have shown significantly elevated concentrations of kynurenine and quinolinic acid, which are closely correlated with cerebrospinal fluid levels of amyloid β1-42, tau protein, and phosphorylated tau-181 protein (Ibáñez et al., 2013). Similar metabolic alterations have been observed in Parkinson’s disease and varicella-zoster virus infections (Trezzi et al., 2017; Kuhn et al., 2018).

To date, the phenotypic spectrum of metabolic abnormalities associated with epilepsy has been characterized by considerable inter-study heterogeneity, and a consensus on the underlying molecular mechanisms is yet to be established. Consequently, reliable biomarkers to aid in the diagnosis of epilepsy are still lacking. Furthermore, only a few metabolomic studies have specifically investigated the relationship between metabolic profile alterations and anti-seizure medication responsiveness in epilepsy, which has limited a deeper understanding of the mechanisms underlying DRE and hindered its early identification and precise intervention. Therefore, this study utilized untargeted metabolomics to systematically reveal metabolic signatures associated with epilepsy and anti-seizure medication responsiveness, screen key candidate metabolites, and provide new clues for precision diagnosis and treatment of epilepsy.

## 2 Materials and methods

### 2.1 Study population

The research adhered to the Declaration of Helsinki and received approval from the Ethics Committee of China-Japan Union Hospital of Jilin University (No. 2023022701). All participants or their authorized representatives provided written informed consent prior to being included in the study.

A total of 42 patients with epilepsy (PWE) were enrolled in this study, including 20 patients diagnosed with DRE and 22 patients with drug-sensitive epilepsy (DSE). In addition, the healthy control (HC) group comprised 20 age- and sex-matched healthy adults. All participants had body mass index values within the normal range and were of Han Chinese origin. To maintain the uniformity and dependability of the study group, the following strict inclusion and exclusion criteria were used: 1) eligible individuals received a formal diagnosis of epilepsy as per the standards set by the International League Against Epilepsy (Fisher et al., 2014); 2) were at least 16 years of age and possessed comprehensive medical records, along with a minimum of 12 months of follow-up data, which included assessments conducted at 3-month intervals; and 3) exhibited a sound mental state sufficient to adhere to the prescribed examinations and treatment protocols. Written informed consent was obtained from the participants or their authorized representatives before inclusion. Participants were deemed ineligible and were subsequently excluded based on the following criteria: 1) any instance in which the follow-up period was inadequate (<12 months), the patient was lost to follow-up, or the patient was <16 years of age; 2) the presence of significant systemic comorbidities such as cardiopulmonary, hepatic, or renal dysfunction; malignancies; diabetes; thyroid disorders; and hematological diseases; and 3) non-standardized antiepileptic treatment, pregnancy or lactation, and a documented history of substance abuse or alcohol dependence.

DRE was defined as failure to achieve sustained seizure freedom, specifically freedom from seizures for at least three times the longest pre-intervention interseizure interval or for a minimum of 12 months, whichever was longer, following adequate trials of two appropriately selected and properly utilized ASM regimens, whether administered as monotherapy or in combination. DSE was defined as freedom from all seizure types for at least 12 months or three times the longest pre-intervention interseizure interval, whichever was longer, irrespective of whether the patient had previously failed 0 or 1 appropriately used ASMs (Kwan et al., 2010; Choi et al., 2020).

### 2.2 Plasma untargeted metabolomics analysis

#### 2.2.1 Plasma sample collection

Venous blood (5 mL) was drawn from fasting participants between 9:00 a.m. and 10:00 a.m. using heparin-coated tubes to control circadian influences. Following collection, the specimens were promptly stored at 4 °C and transported to the biorepository at the China-Japan Union Hospital of Jilin University within 60 min for plasma isolation. The plasma obtained was divided into smaller samples and frozen at −80 °C until it was used for metabolomic profiling.

#### 2.2.2 Plasma samples preparation

Plasma samples were prepared for metabolomic profiling by using a stepwise extraction method. Briefly, 100 µL of plasma was mixed with four volumes of ice-cold methanol/acetonitrile (1:1, v/v) and thoroughly vortexed. The mixture was sonicated on ice to enhance protein precipitation and metabolite release, followed by incubation at −20 °C. After centrifuging at 14,000 g and 4 °C, the supernatant was gathered and vacuum-dried. The dried residues were subsequently subjected to ultra-high performance liquid chromatography-tandem mass spectrometry (UHPLC-MS/MS).

#### 2.2.3 UHPLC-MS/MS analysis

Metabolomic profiling was performed using a Shimadzu Nexera X2 UHPLC coupled with a Q-Exactive Plus mass spectrometer. The separation utilized an ACQUITY UPLC BEH Amide column (2.1 × 100 mm, 1.7 µm) with a flow rate of 0.5 mL/min under hydrophilic interaction liquid chromatography conditions. MS data were collected using both positive and negative ionization modes. In positive ion mode, the spray voltage was maintained at 3.8 kV, while in negative ion mode it was set to 3.2 kV. Additional instrumental parameters were configured as follows: capillary temperature, 320 °C; sheath gas flow, 30 arbitrary units; auxiliary gas flow, 5 arbitrary units; probe heater temperature, 350 °C; and S-lens RF level, 50. Full MS scans were acquired over an m/z range of 80–1,200 at a resolution of 70,000 (m/z 200), with a maximum injection time of 100 ms. MS/MS scans were acquired at a resolution of 17,500 (m/z 200) with a 2 m/z isolation window, stepped collision energies of 27, 29, and 32, and a maximum injection time of 50 ms. Quality control (QC) samples were included during acquisition. A pooled QC sample was prepared by combining equal aliquots of plasma samples, and eight QC injections were analyzed during LC–MS acquisition to monitor analytical stability and reproducibility. The QC samples clustered tightly in principal component analysis, and the chromatographic peak intensities and retention times of the QC injections were generally consistent in both positive- and negative-ion modes. The majority of detected ion features exhibited QC relative standard deviations below 30%.

### 2.3 Data preprocessing

The raw mass spectrometry data from the PWE and HC groups underwent processing in MS-DIAL for aligning peaks, correcting retention times, and integrating peak areas. Metabolite annotation was performed by matching precursor exact mass (mass tolerance <0.01 Da) and MS/MS fragment spectra (mass tolerance <0.02 Da) against the Human Metabolome Database, MassBank, other public databases, and an in-house metabolite standard library. Only features detected in more than 50% of samples in at least one group were retained for downstream analysis.

### 2.4 Statistical analysis

Continuous variables, presented as median (P25, P75), were compared via the Kruskal-Wallis test, whereas categorical variables, shown as n (%), were assessed with the χ^2^ test or Fisher’s exact test.

Welch’s t-test and fold change (FC) analyses were initially performed, and the results were visualized using volcano plots. For false discovery rate correction, we used the Benjamini-Hochberg method. Multivariate statistical approaches, including principal component analysis (PCA), partial least squares discriminant analysis (PLS-DA), and orthogonal partial least squares discriminant analysis (OPLS-DA), were performed to characterize the group separation. Model validity was assessed using a 200-iteration permutation test. The differential metabolites were further analyzed using the Kyoto Encyclopedia of Genes and Genomes (KEGG) database (https://www.kegg.jp/) to identify the principal metabolic pathways affected. Next, key differential metabolites were selected by applying the least absolute shrinkage and selection operator (LASSO) regression together with support vector machine (SVM) algorithms and random forest (RF). Receiver operating characteristic (ROC) curves and violin plots were employed to visualize the identified metabolites. Binary logistic regression models were subsequently established based on the candidate metabolites. Given the relatively small sample size, internal validation was performed using the bootstrap method with 1,000 resamples. The model’s evaluation included accuracy, sensitivity, specificity, predictive values, area under the curve (AUC), calibration, and decision curve analysis. All analyses were performed using R software (version 4.5.1).

### 2.5 Integrated analysis of transcriptomics and untargeted metabolomics

Transcriptomic data were retrieved from the Gene Expression Omnibus database (https://www.ncbi.nlm.nih.gov/geo/) under accession GSE256068, a human bulk RNA-sequencing dataset generated on the Illumina NovaSeq 6000 platform. For the present analysis, 64 human temporal lobe epilepsy with hippocampal sclerosis samples and 13 control hippocampal samples were selected from this dataset, and the processed count matrix provided by the Gene Expression Omnibus database was used for downstream analysis. Differential expression analysis was performed to identify differentially expressed genes (DEGs) between the two groups, followed by KEGG pathway enrichment analysis. To integrate the transcriptomic and untargeted metabolomic data, the KEGG-enriched pathways derived from DEGs were intersected with those identified from differential metabolites. The shared pathways were regarded as convergent pathways at the transcriptomic and metabolomic level. Additionally, a gene–metabolite–pathway network was constructed. Figure 1 illustrates the study’s workflow.

**FIGURE 1:**
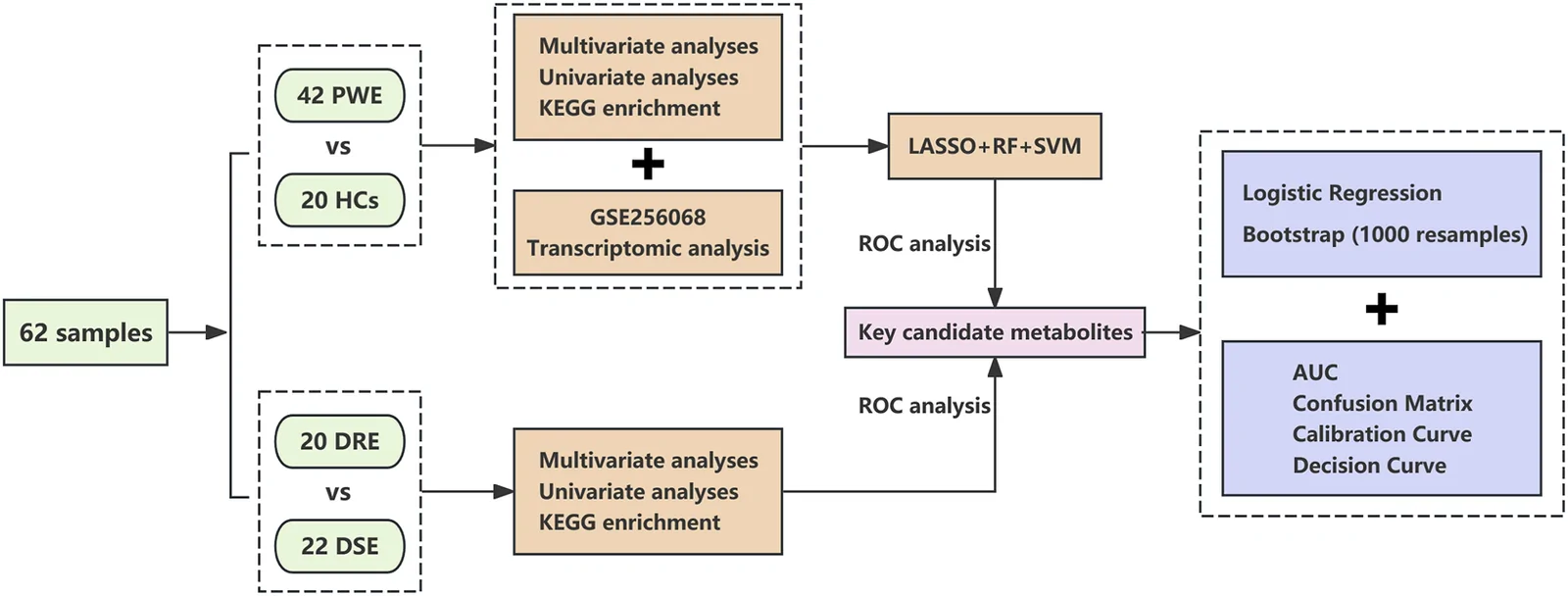
Overall workflow of the study.

## 3 Results

### 3.1 Study cohort and sample characteristics

In total, 42 PWE were included in this study and were assigned to the DRE (n = 20) and DSE (n = 22) subgroups, with 20 healthy individuals serving as controls. In Table 1, the baseline characteristics of the participants are outlined, with age and sex showing no significant differences among the groups. No significant differences were observed between the DRE and DSE groups in seizure classification or epilepsy duration, whereas the DRE group had higher proportions of polytherapy, abnormal brain magnetic resonance imaging findings, and structural etiology.

**TABLE 1:** Demographic and clinical characteristics of enrolled participants.

Demographic characteristics	Total	Group			
		HCs (n = 20)	DRE (n = 20)	DSE (n = 22)	P value
Age, y, median (IQR)	27.5 (23.75–35.5)	27 (25–30)	30 (23–51.75)	28 (19–38)	0.585^a^
Male, n (%)	37 (60%)	10 (50%)	14 (70%)	13 (59%)	0.434^b^
Seizure classification, n (%)					
Focal	38 (90.5%)		20 (100%)	18 (81.8%)	0.109^c^
Generalized	4 (9.5%)		0 (0.0%)	4 (18.2%)	
Epilepsy duration, n (%)					
≤5 years	22 (52.4%)		9 (45.0%)	13 (59.1%)	0.361^b^
>5 years	20 (47.6%)		11 (55.0%)	9 (40.9%)	
ASM treatment pattern, n (%)					
Monotherapy	30 (71.4%)		10 (50.0%)	20 (90.9%)	0.003^b^
Polytherapy	12 (28.6%)		10 (50.0%)	2 (9.1%)	
Abnormal brain MRI, n (%)					
Yes	28 (66.7%)		20 (100%)	8 (36.4%)	<0.001^b^
No	14 (33.3%)		0 (0.0%)	14 (63.6%)	
Etiology					
Structural	26 (61.9%)		19 (95.0%)	7 (31.8%)	<0.001^c^
Genetic	4 (9.5%)		0 (0.0%)	4 (18.2%)	
Infectious	1 (2.4%)		0 (0.0%)	1 (4.5%)	
Unknown	11 (26.2%)		1 (5.0%)	10 (45.5%)	

^a^ P value calculated using Kruskal-Wallis tests.

^b^ P value calculated using χ2 test.

^c^ P value calculated using Fisher’s exact test. ASM, Anti-seizure medication; HCs, Healthy controls; DRE, Drug-resistant epilepsy; DSE, Drug-sensitive epilepsy; MRI, magnetic resonance imaging; IQR, interquartile range.

### 3.2 Identification of metabolic signatures between PWE and HC groups

#### 3.2.1 PCA, PLS-DA, and OPLS-DA model construction

The results indicated that the PCA scores plot showed a modest separation between the PWE and HC groups (Figure 2A). In the supervised models, group discrimination was further enhanced, and the OPLS-DA scores plot demonstrated clearer clustering between the two groups (Figures 2B,C). Permutation testing indicated that the PLS-DA model had a positive Q2 regression intercept (0.54), suggesting that its interpretation should be approached with caution, whereas the OPLS-DA model had a negative Q2 regression intercept (−0.219), supporting its better stability (Figures 2D,E).

**FIGURE 2:**
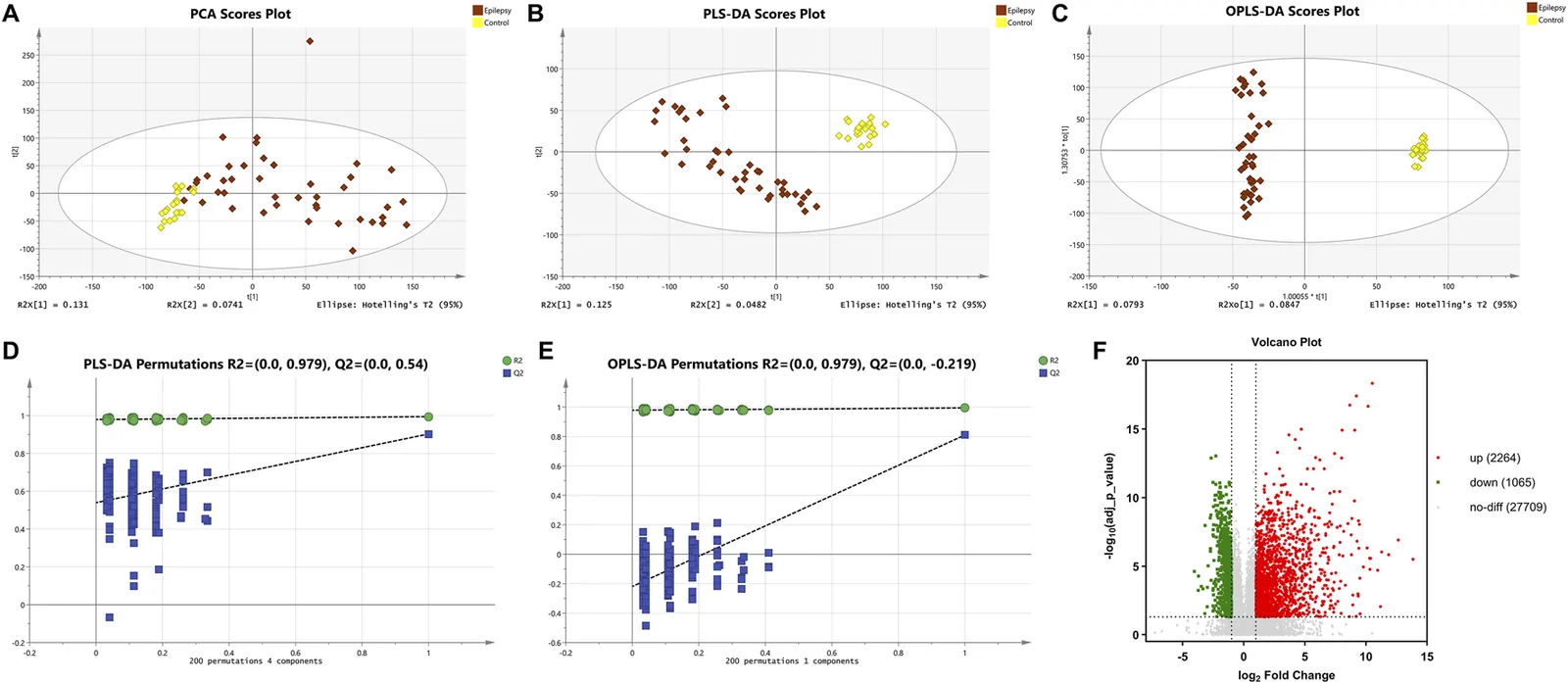
Multivariate and univariate statistical analyses between the PWE and HC groups. **(A)** PCA scores plot. **(B)** PLS-DA scores plot. **(C)** OPLS-DA scores plot. **(D)** Permutation test (200 iterations) for the PLS-DA model. **(E)** Permutation test (200 iterations) for the OPLS-DA model. **(F)** Volcano plot of differential ion features identified using adjusted P value <0.05 and |log_2_FC| > 1. PCA, principal component analysis; PLS-DA, partial least squares discriminant analysis; OPLS-DA, orthogonal partial least squares discriminant analysis; FC, fold change; PWE, patients with epilepsy; HC, healthy control.

#### 3.2.2 Analysis of differential metabolic features

Untargeted metabolomics detected a total of 31,038 features, of which 914 were annotated as metabolites. Welch’s t-test combined with FC analysis was used to compare the PWE and HC groups, and the results were visualized in a volcano plot. Using the thresholds of adjusted P value < 0.05 and |log2FC| > 1, we identified 3,329 significantly altered features, including 2,264 increased and 1,065 decreased features in the PWE group relative to the HC group (Figure 2F).

#### 3.2.3 KEGG enrichment analysis

KEGG enrichment analysis results showed that these differential metabolites were mainly annotated to metabolism-related pathways, human disease-related pathways, and organismal system-related pathways (Figure 3A). KEGG enrichment analysis identified the taurine and hypotaurine metabolism pathway, vitamin digestion and absorption pathway, tyrosine metabolism pathway, cholesterol metabolism pathway, cyclic adenosine monophosphate (cAMP) signaling pathway, hypoxia-inducible factor-1 (HIF-1) signaling pathway, and dopaminergic synapse pathway as enriched (Figure 3B; Supplementary Table S1). Analysis of the transcriptomic dataset showed clear transcriptional differences between the PWE and HC groups. The bubble plot of shared KEGG pathways further demonstrated that DEGs and differential metabolites converged on multiple common pathways, including the cAMP signaling pathway, HIF-1 signaling pathway, and dopaminergic synapse pathway (Figure 3C).

**FIGURE 3:**
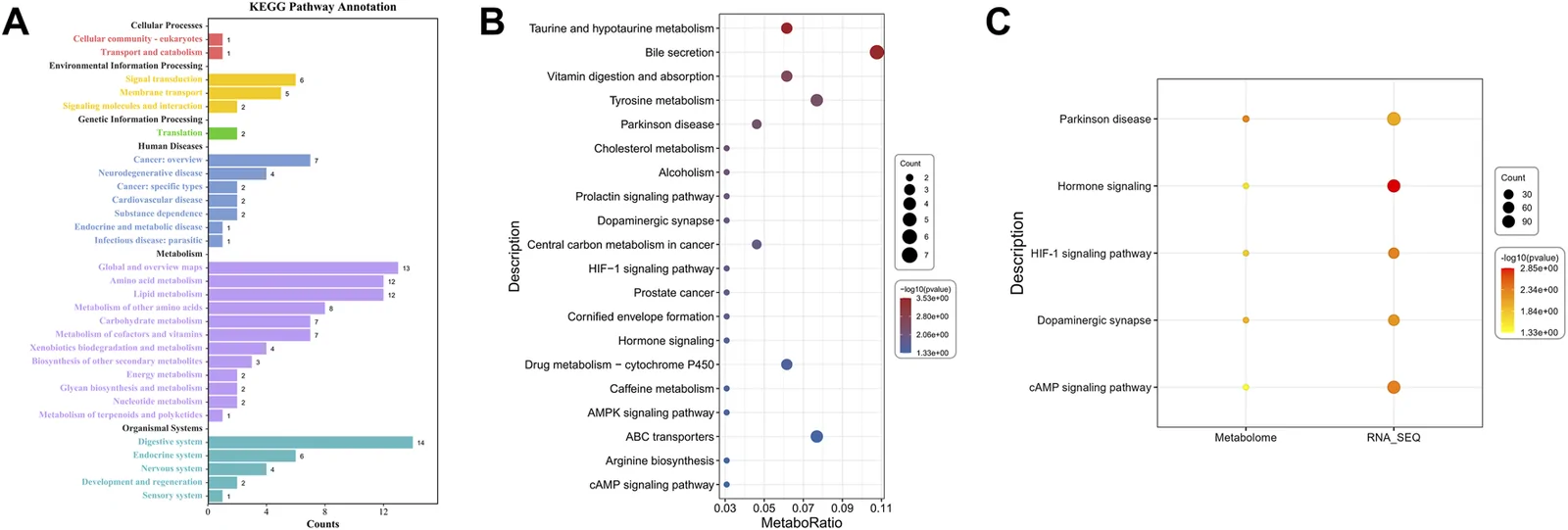
KEGG enrichment analysis of differential metabolites between the PWE and HC groups. **(A)** Differential metabolites KEGG classification map. Bar length represents the number of metabolites assigned to each KEGG subcategory. **(B)** Differential metabolites KEGG enrichment pathway map. Bubble size indicates the number of mapped metabolites, and color represents −log10 (P value). **(C)** Bubble plot of shared KEGG pathways between the metabolomic and transcriptomic analyses. Bubble size indicates the number of mapped metabolites or DEGs, as applicable, and color represents −log10 (P value). DEGs, differentially expressed genes; KEGG, Kyoto Encyclopedia of Genes and Genomes; PWE, patients with epilepsy; HC, healthy control.

To further characterize these cross-omics associations, we constructed a gene–metabolite–pathway network based on three key KEGG pathways. The cAMP signaling pathway, HIF-1 signaling pathway, and dopaminergic synapse pathway contained 102, 54, and 63 DEGs, respectively, yielding a total of 186 unique DEGs after removing genes shared across pathways (Supplementary Table S1). Based on the pathway membership of these DEGs across the three key pathways, we further classified them into seven mutually exclusive pathway-membership categories: DEGs belonging exclusively to the cAMP signaling pathway, exclusively to the HIF-1 signaling pathway, exclusively to the dopaminergic synapse pathway, jointly to the cAMP and HIF-1 signaling pathways, jointly to the cAMP signaling pathway and dopaminergic synapse pathway, jointly to the HIF-1 signaling pathway and dopaminergic synapse pathway, and jointly to all three pathways. Further analysis showed that 73, 39, and 44 DEGs belonged exclusively to the cAMP signaling pathway, HIF-1 signaling pathway, and dopaminergic synapse pathway, respectively. In addition, 11, 15, and 1 DEGs were shared between the cAMP and HIF-1 signaling pathways, between the cAMP signaling pathway and dopaminergic synapse pathway, and between the HIF-1 signaling pathway and dopaminergic synapse pathway, respectively, while three DEGs were shared across all three pathways. The network constructed on the basis of these pathway-membership relationships further linked differential metabolites and DEGs through the cAMP signaling pathway, HIF-1 signaling pathway, and dopaminergic synapse pathway (Supplementary Figure S1). Overall, these results indicate that epilepsy-associated molecular alterations involve both the transcriptomic and metabolomic levels and converge on several biologically relevant pathways.

#### 3.2.4 Identification of key candidate metabolites by machine learning

By integrating multivariate statistical analysis (variable importance in projection score >1) with univariate statistical analysis (|log2FC| > 1 and adjusted P value < 0.05), we identified 122 annotated differential metabolites. Before machine-learning analysis, the annotated metabolites underwent a secondary review based on chemical source, biological plausibility in human plasma, and annotation specificity. Known drugs and drug metabolites; contaminants related to environmental, industrial, or agrochemical exposure; dietary, nutrient, or plant-derived compounds; and annotations considered chemically ambiguous or insufficiently specific for biological interpretation were excluded, leaving 16 candidate metabolites for subsequent analysis (Supplementary Table S2). LASSO regression, SVM, and RF were then independently applied for feature selection and prioritization (Figures 4A–E). The intersection of the three machine-learning methods identified three key candidate metabolites: palmitoylcarnitine, C18 (plasm)-18:1 PC, and 1-palmitoyl-2-oleoyl-sn-glycero-3-phosphocholine (Figure 4F).

**FIGURE 4:**
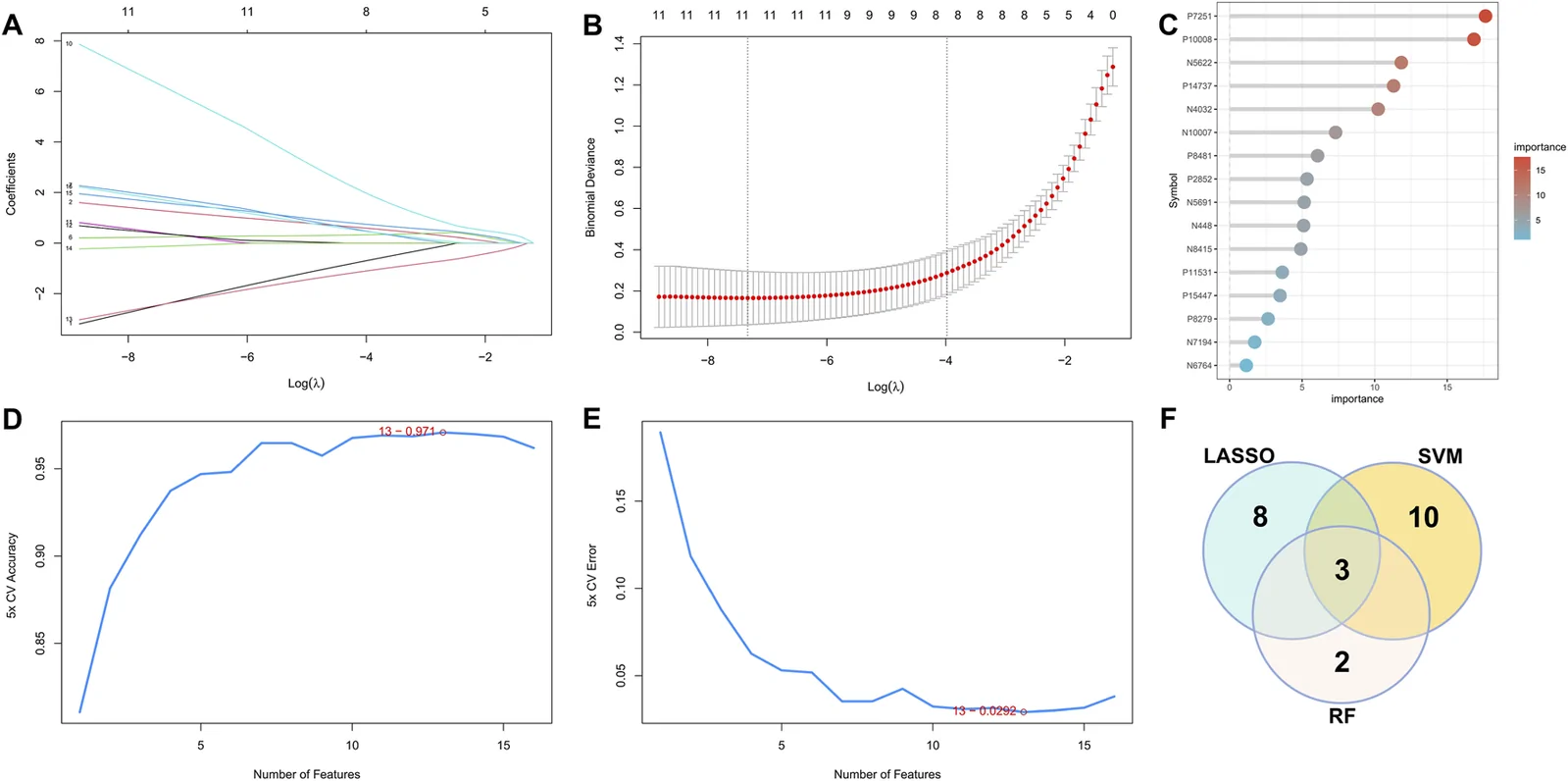
Identification of key candidate metabolites between the PWE and HC groups using LASSO regression, RF, and support vector machine (SVM) algorithms. **(A)** LASSO coefficient path plot. Each curve represents the process by which a variable’s coefficient is gradually compressed to zero as the penalty strength increases. **(B)** Cross-validation curve for LASSO regression. It shows the prediction error of the model under different penalty strengths, with the lowest point corresponding to λmin, which is the model with the smallest error. **(C)** RF feature-importance ranking. Higher importance values indicate a greater contribution to RF classification. **(D,E)** SVM-based feature selection process. Panel D shows cross-validation accuracy, and panel E shows the corresponding cross-validation error across different numbers of retained features. **(F)** Overlap of metabolites identified by LASSO regression, RF, and SVM. PWE, patients with epilepsy; HC, healthy control; LASSO, least absolute shrinkage and selection operator; RF, random forest; SVM, support vector machine.

To evaluate the individual discriminative performance of these metabolites, ROC curves and violin plots were generated. Palmitoylcarnitine, C18 (plasm)-18:1 PC, and 1-palmitoyl-2-oleoyl-sn-glycero-3-phosphocholine yielded AUCs of 0.929 (95% CI: 0.867–0.991), 0.886 (95% CI: 0.797–0.974), and 0.876 (95% CI: 0.792–0.961), respectively (all P < 0.0001). Palmitoylcarnitine and 1-palmitoyl-2-oleoyl-sn-glycero-3-phosphocholine were increased in the PWE group, whereas C18 (plasm)-18:1 PC was decreased (Figure 5).

**FIGURE 5:**
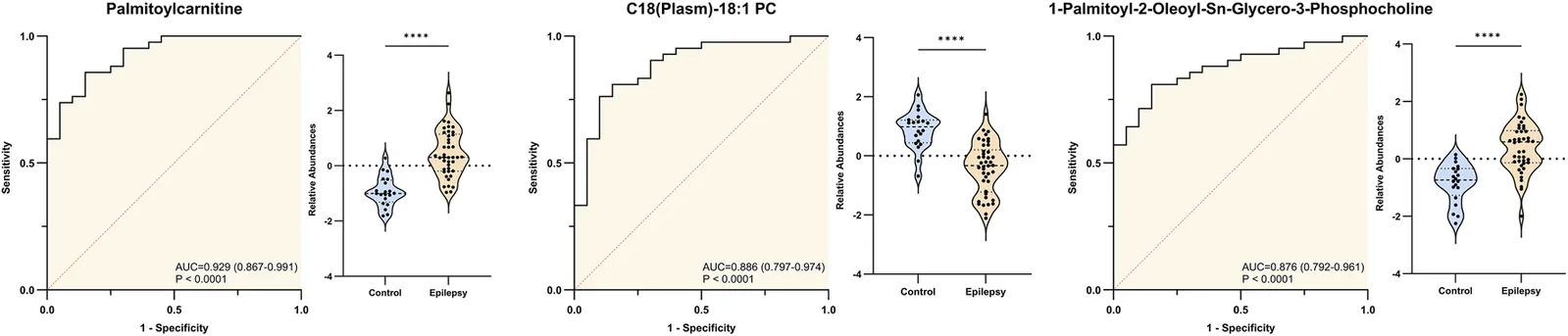
ROC curve analysis and violin plots of the three key candidate metabolites distinguishing the PWE and HC groups. ROC curves show the individual discriminatory performance, whereas violin plots show the relative abundance distributions between groups. ROC, receiver operating characteristic; PWE, patients with epilepsy; HC, healthy control.

#### 3.2.5 Logistic regression model construction and evaluation

Using the three key candidate metabolites, we developed a binary logistic regression model to discriminate the PWE group from the HC group and assessed its internal performance using 1,000 bootstrap resamples. ROC analysis showed an apparent AUC of 0.989 (95% CI: 0.968–1.000). After bootstrap-based optimism correction, the corrected AUC derived from the Dxy statistic was 0.987, while the optimism-corrected ROC curve yielded an AUC of 0.973 (Figure 6A). The confusion matrix identified 41 true positives, 19 true negatives, 1 false positive, and 1 false negative, corresponding to an accuracy of 96.8% (60/62), a sensitivity of 97.6% (41/42), a specificity of 95.0% (19/20), a positive predictive value of 97.6% (41/42), and a negative predictive value of 95.0% (19/20) (Figure 6B). Bootstrap calibration analysis yielded a mean absolute error of 0.085 (Figure 6C). Decision curve analysis showed that the model provided a greater net benefit than the treat-all and treat-none strategies across a broad range of threshold probabilities (Figure 6D).

**FIGURE 6:**
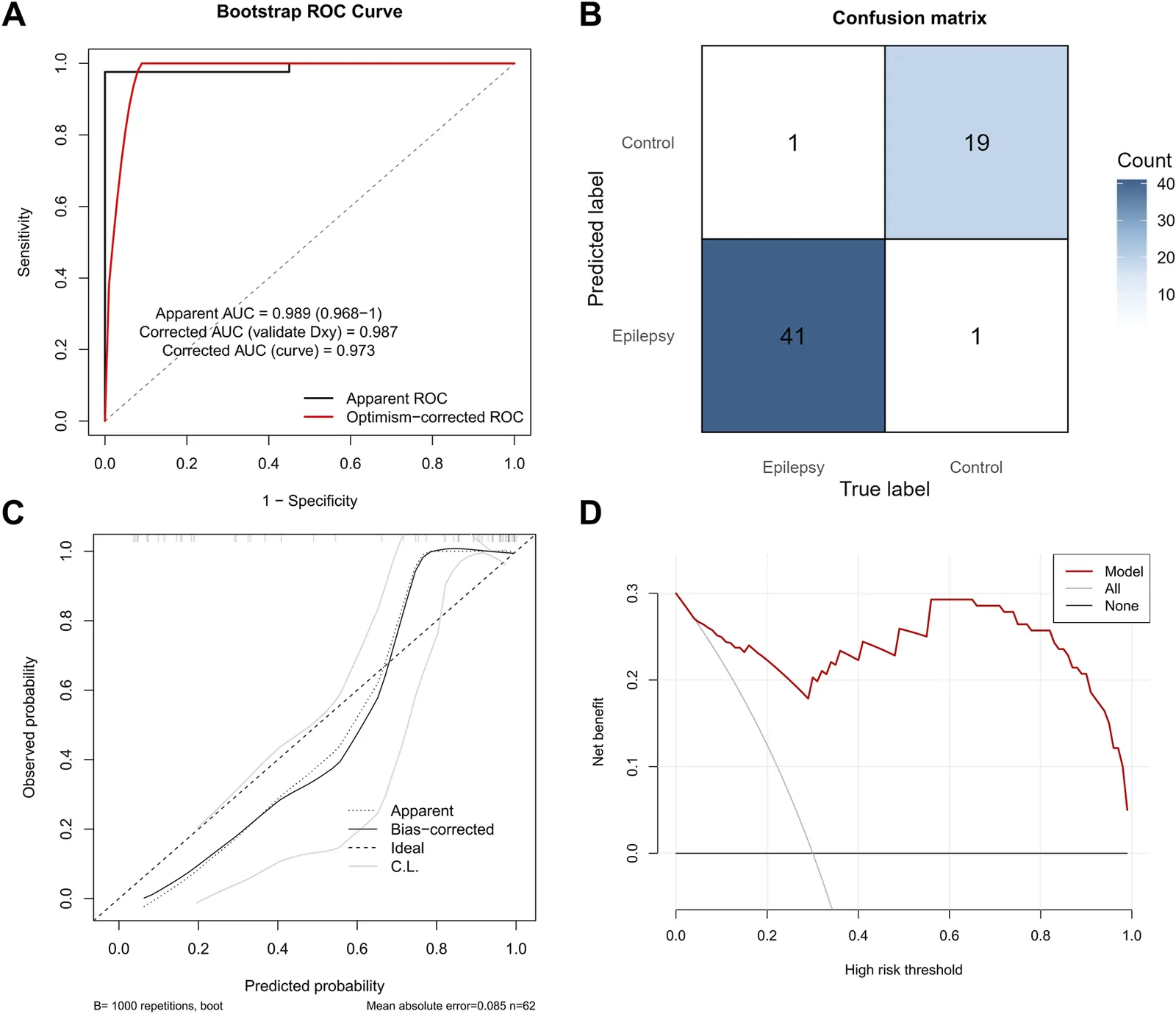
Construction and performance evaluation of the binary logistic regression model differentiating the PWE group from the HC group. **(A)** ROC curve. The black curve represents apparent model performance and the red curve represents bootstrap-based optimism-corrected performance. **(B)** Confusion matrix. Rows represent predicted labels and columns represent true labels, with cell values indicating sample counts. **(C)** Calibration curve. Apparent and bootstrap bias-corrected calibration are compared with the ideal reference line. **(D)** Decision curve analysis. Net benefit is evaluated across threshold probabilities relative to the treat-all and treat-none strategies. PWE, patients with epilepsy; HC, healthy control; ROC, receiver operating characteristic.

### 3.3 Identification of metabolic signatures between DRE and DSE groups

#### 3.3.1 Intergroup comparisons and KEGG enrichment analysis

We further explored differences in plasma metabolic profiles between patients with DRE and those with DSE. Given the relatively modest metabolic differences between the DRE and DSE groups and the limited sample size of this subgroup analysis, the analysis was considered exploratory, and relatively less stringent thresholds were adopted for differential feature screening. First, an OPLS-DA model was constructed, followed by permutation testing to assess model validity (Figures 7A,B). Permutation testing indicated limited predictive robustness and potential overfitting, suggesting that the OPLS-DA model was not sufficiently robust for reliable differential feature identification. We therefore performed univariate analysis as an alternative approach. Using the thresholds of |log2FC| > 0.2 and P < 0.05, we identified 1,074 differential ion features between the DRE and DSE groups, including 485 upregulated and 589 downregulated features (Figure 7C). KEGG enrichment analysis showed that the differential metabolites were primarily enriched in tryptophan metabolism, the citrate cycle (TCA cycle), and alanine, aspartate and glutamate metabolism (Supplementary Figure S2; Supplementary Table S1).

**FIGURE 7:**
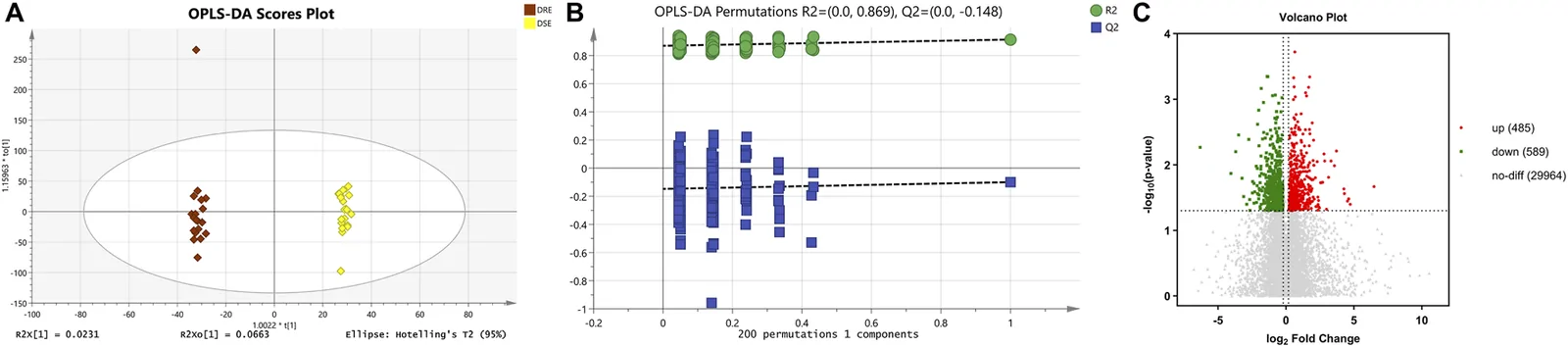
Multivariate and univariate statistical analyses between the DRE and DSE groups. **(A)** OPLS-DA scores plot. **(B)** Permutation test (200 iterations) for the OPLS-DA model. **(C)** Volcano plot of differential ion features identified using |log2FC| > 0.2 and P < 0.05. DRE, drug-resistant epilepsy; DSE, drug-sensitive epilepsy; OPLS-DA, orthogonal partial least squares discriminant analysis; FC, fold change.

#### 3.3.2 Identification of key candidate metabolites

Of these 1,074 differential ion features, 39 were annotated as metabolites. After excluding known drugs and drug metabolites; contaminants related to environmental, industrial, or agrochemical exposure; dietary, nutrient, or plant-derived compounds; and annotations considered chemically ambiguous or insufficiently specific for biological interpretation, seven candidate metabolites were retained: L-kynurenine, indolelactate, glycodeoxycholic acid, N-acetylcytidine, citric acid, 5-hydroxyindoleacetate, and myristic acid (Supplementary Table S3). ROC analysis showed that six of these seven metabolites significantly discriminated DRE from DSE, with AUCs ranging from 0.677 to 0.741 (all P < 0.05), whereas myristic acid did not reach statistical significance (AUC = 0.664, P = 0.0698). Relative to the DSE group, the DRE group showed lower levels of L-kynurenine, indolelactate, N-acetylcytidine, citric acid, and 5-hydroxyindoleacetate, but higher levels of glycodeoxycholic acid, supporting their discriminatory potential between the DRE and DSE groups (Figure 8).

**FIGURE 8:**
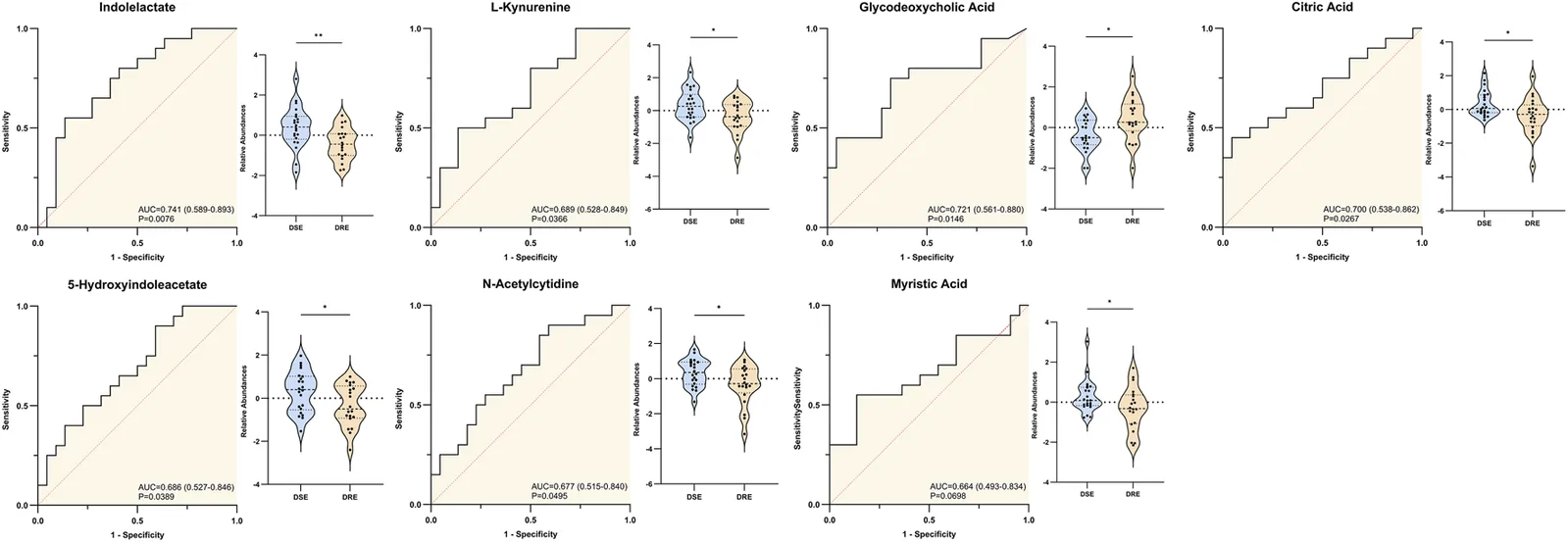
ROC curve analysis and violin plots of the seven candidate metabolites between the DRE and DSE groups. ROC curves show individual discriminatory performance, whereas violin plots show the relative abundance distributions between groups. DRE, drug-resistant epilepsy; DSE, drug-sensitive epilepsy; ROC, receiver operating characteristic.

#### 3.3.3 Logistic regression model construction and evaluation

Using the six key candidate metabolites, we developed a binary logistic regression model to discriminate DRE from DSE and assessed its internal validity by 1,000 bootstrap resamples. The model showed an apparent AUC of 0.866 (95% CI: 0.760–0.971). After optimism correction, the Dxy-derived corrected AUC was 0.785, and the optimism-corrected ROC curve yielded an AUC of 0.773 (Figure 9A). The confusion matrix identified 15 true positives, 17 true negatives, 5 false positives, and 5 false negatives, resulting in an accuracy of 76.2% (32/42), a sensitivity of 75.0% (15/20), a specificity of 77.3% (17/22), a positive predictive value of 75.0% (15/20), and a negative predictive value of 77.3% (17/22) (Figure 9B). Bootstrap calibration analysis yielded a mean absolute error of 0.094 (Figure 9C), while decision curve analysis indicated that the model provided a greater net benefit than the treat-all and treat-none strategies across most threshold probabilities (Figure 9D).

**FIGURE 9:**
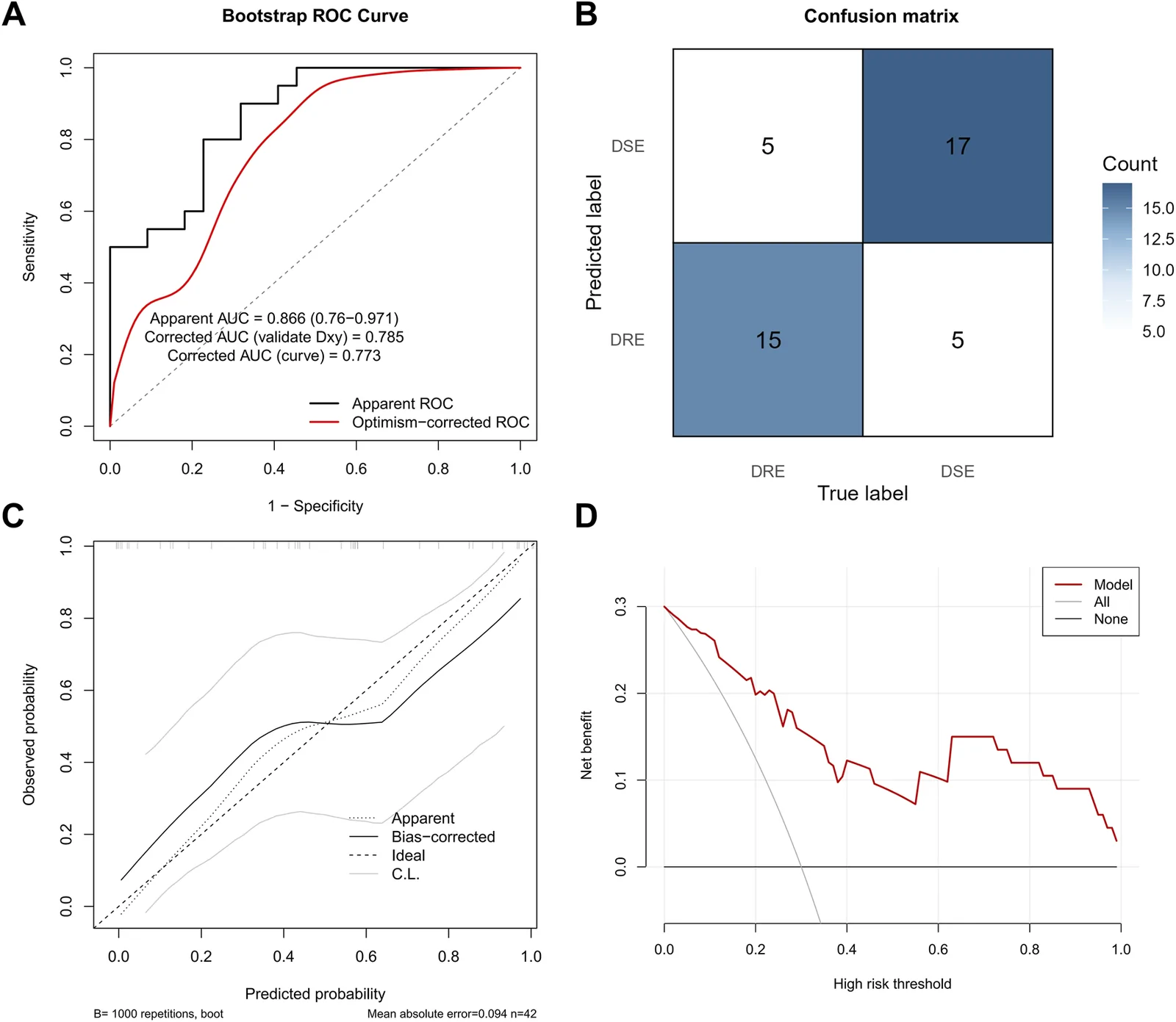
Construction and performance evaluation of the binary logistic regression model differentiating the DRE group from the DSE group. **(A)** ROC curve. The black curve represents apparent model performance and the red curve represents bootstrap-based optimism-corrected performance. **(B)** Confusion matrix. Rows represent predicted labels and columns represent true labels, with cell values indicating sample counts. **(C)** Calibration curve. Apparent and bootstrap bias-corrected calibration are compared with the ideal reference line. **(D)** Decision curve analysis. Net benefit is evaluated across threshold probabilities relative to the treat-all and treat-none strategies. DRE, drug-resistant epilepsy; DSE, drug-sensitive epilepsy; ROC, receiver operating characteristic.

## 4 Discussion

This study systematically characterized the metabolic profiles of PWE and identified key candidate metabolic features associated with epilepsy and anti-seizure medication responsiveness. The results indicated that the cAMP signaling pathway, the HIF-1 signaling pathway, and the dopaminergic synapse pathway may be involved in the pathological mechanisms of epilepsy. With respect to discriminatory performance, three metabolites—palmitoylcarnitine, C18 (plasm)-18:1 PC, and 1-palmitoyl-2-oleoyl-sn-glycero-3-phosphocholine—effectively distinguished PWE from HCs, whereas L-kynurenine, indolelactate, glycodeoxycholic acid, N-acetylcytidine, citric acid, and 5-hydroxyindoleacetate showed potential discriminatory value between the DRE and DSE groups.

The cAMP signaling pathway has been implicated in epilepsy-related pathophysiology. Activation of this pathway depends on the binding of neurotransmitters such as dopamine to G protein-coupled receptors, which subsequently activates adenylyl cyclase (AC), catalyzing the conversion of ATP to cAMP. cAMP subsequently activates protein kinase A (PKA), which results in the phosphorylation of the transcription factor known as cAMP response element-binding protein (CREB). Phosphorylated CREB, in turn, promotes the expression of the transcription factor cellular fos proto-oncogene, thereby contributing to hippocampal neuronal death and, consequently, to epileptogenesis and disease progression (Gautam et al., 2021). Jiang et al. (2018) demonstrated that the cAMP signaling pathway is significantly enriched in the hippocampal CA3 region of epilepsy patients, involving key genes such as CALM1, GRIA1, and CAMK2B. Furthermore, the glutamate receptor 2 participates in the cAMP signaling pathway, and its expression is markedly reduced in the hippocampus of epileptic rats. Treatment with quinpirole reverses the decrease in glutamate receptor 2 levels, hence restoring the balance between excitatory and inhibitory neurotransmission (Wang et al., 2024). The dopaminergic synapse signaling pathway modulates seizure susceptibility through D1-like and D2-like receptors. Specifically, D1 receptors couple to Gs proteins and activate AC, thereby elevating cAMP/PKA signaling and promoting the phosphorylation of extracellular signal-regulated kinase, consequently facilitating neuronal firing. Accordingly, D1 receptor agonists reduce the seizure threshold, whereas mice lacking D1 receptors exhibit attenuated seizure severity. In contrast, D2 receptors couple to Gi proteins and inhibit AC; therefore, D2 receptor activation exerts anticonvulsant effects, while D2 receptor blockade or genetic deletion increases seizure susceptibility (Bozzi and Borrelli, 2013). Moreover, the HIF-1 signaling pathway is closely associated with epilepsy. Liang et al. demonstrated that the HIF-1 pathway is significantly enriched in the peripheral blood of epilepsy patients, and HIF-1α may represent a potential therapeutic target for epilepsy. In the pentylenetetrazol-kindled mouse model of epilepsy, the expression levels of hippocampal HIF-1α and its downstream target gene heme oxygenase-1 were markedly elevated, accompanied by abnormal iron metabolism and Fe^2+^ accumulation, consequently inducing ferroptosis in hippocampal neurons (Liang et al., 2023).

This study suggests a potential association between abnormalities in lipid metabolism and epilepsy. Palmitoylcarnitine is a long-chain acylcarnitine and an important intermediate in the carnitine shuttle, through which long-chain fatty acids enter mitochondria for β-oxidation; its accumulation generally reflects impaired fatty acid oxidation. Previous studies have shown that the accumulation of long-chain acylcarnitines can exacerbate mitochondrial injury in astrocytes and impair their metabolic support for neurons. In addition, palmitoylcarnitine has been reported to induce oxidative stress, lipid peroxidation, calcium overload, and mitochondrial dysfunction (Huang et al., 2024; Eswaran et al., 2025). Therefore, the elevated palmitoylcarnitine observed in this study may indicate impaired fatty acid oxidation and disrupted mitochondrial energy metabolism in patients with epilepsy. However, the above evidence is mainly derived from other disease models, and the direct role of palmitoylcarnitine in epilepsy remains to be further validated. C18 (plasm)-18:1 PC and 1-palmitoyl-2-oleoyl-sn-glycero-3-phosphocholine are both membrane phospholipid-related molecules. Phospholipids are major structural components of cell membranes and contribute to the maintenance of membrane integrity and cellular signaling homeostasis (Kang J. H. et al., 2025). Notably, a lipid imaging study of the hippocampus in patients with temporal lobe epilepsy reported reduced levels of phosphatidylcholine and phosphatidylethanolamine and suggested potential dysregulation of the Kennedy pathway (Ajith et al., 2021). The two phospholipid molecules identified in the present study showed changes in opposite directions, which may be more indicative of alterations in membrane phospholipid composition and remodeling in epilepsy rather than a unidirectional change in overall phospholipid levels. However, direct evidence that these two phospholipid molecules participate in the pathophysiological mechanisms of epilepsy is currently lacking; therefore, these findings should be considered exploratory and suggestive.

L-kynurenine is generated through the oxidative cleavage of tryptophan catalyzed by indoleamine 2,3-dioxygenase 1 (Kaur et al., 2025). Studies have shown that quinolinic acid, a downstream metabolite of L-kynurenine, can activate N-methyl-D-aspartate receptors and thereby enhance glutamate excitotoxicity (Kaur et al., 2025). In the present study, L-kynurenine levels were decreased in the DRE group, suggesting dysregulation of kynurenine metabolism; however, the relationship between its downstream neurotoxic metabolites and drug resistance in epilepsy remains to be established. Although direct experimental evidence linking indolelactate to DRE is currently lacking, studies in multiple sclerosis and autism spectrum disorder have demonstrated that this metabolite possesses significant anti-inflammatory and neuroprotective potential (Levi et al., 2021; Aziz-Zadeh et al., 2025). Hence, the decreased indolelactate levels observed in patients with DRE may reflect impairment of the protective metabolic axis associated with indolelactate. Glycodeoxycholic acid is a conjugated secondary bile acid formed by the conjugation of deoxycholic acid (DCA) with glycine in the liver. Currently, there is no direct evidence implicating glycodeoxycholic acid in drug resistance in epilepsy. Research has demonstrated that bile acids can disrupt blood-brain barrier integrity and activate the nuclear factor kappa B (NF-κB) pathway, thereby promoting neuroinflammation (Chen et al., 2022; Zhang et al., 2026). However, whether glycodeoxycholic acid indirectly influences the development of drug resistance in epilepsy by exacerbating neuroinflammation and blood-brain barrier dysfunction remains to be further experimentally validated. N-acetylcytidine is an acetylated nucleoside that can affect RNA stability and translational efficiency (Bartova et al., 2025). In the present study, plasma N-acetylcytidine levels were decreased in patients with DRE, implying that DRE may involve alterations in nucleoside metabolism or RNA modification-related processes. Citric acid is an important intermediate of the tricarboxylic acid cycle and is closely associated with mitochondrial energy metabolism. A previous metabolomic study in children with DRE similarly reported decreased serum citrate levels and alterations in the tricarboxylic acid cycle (Boguszewicz et al., 2019). The decreased citric acid levels observed in the DRE group in the present study further suggest that DRE may be accompanied by alterations in mitochondrial energy metabolism; however, whether citric acid directly contributes to drug resistance in epilepsy remains uncertain. 5-Hydroxyindoleacetate is a metabolite of serotonin and can reflect the status of serotonin metabolism (Mujagic et al., 2022). Although direct evidence linking 5-hydroxyindoleacetate to epilepsy is currently lacking, its decreased level observed in the present study provides an important clue to DRE-associated alterations in serotonin metabolism.

The binary logistic regression model constructed in this study demonstrated favorable discriminatory performance in distinguishing PWE from HCs, and achieved a moderate level of accuracy in differentiating DRE from DSE. These findings suggest that combinations of peripheral blood metabolites may hold potential clinical value as auxiliary tools for epilepsy diagnosis and for assessing drug responsiveness. Previous studies have made notable contributions to the exploration of metabolic biomarkers in epilepsy. For instance, Kang W. et al. (2025) developed an early predictive model for DRE based on serum untargeted metabolomics, revealing significant perturbations in pathways such as sphingolipid metabolism and choline metabolism; their SVM model yielded an AUC of 0.7437 in an independent test set. Lian et al. (2024) constructed a diagnostic model using four serum metabolites that effectively distinguished patients experiencing a first seizure from healthy individuals, achieving an AUC of 100% in the validation set. Detour et al. (2018), meanwhile, focused on metabolic heterogeneity across different hippocampal pathological subtypes, underscoring the relevance of localized metabolic differences for epilepsy classification. In comparison, our study not only compared plasma metabolic profiles between PWE and HC groups, but also further stratified patients into DSE and DRE subgroups based on long-term follow-up data. Moreover, this study employed a high-resolution UHPLC–MS/MS platform with rigorous quality control procedures. The metabolite selection process integrated multivariate and univariate statistical analyses, as well as machine-learning-based feature selection, thereby effectively reducing model complexity and the risk of overfitting. Furthermore, calibration curves and decision curve analysis were incorporated into the model evaluation framework, thereby enhancing both interpretability and clinical translational potential.

Despite the positive outcomes, this study has several limitations. First, because PWE were receiving ASMs, whereas HCs were not, the present study could not fully distinguish disease-related metabolic alterations from medication-related effects. Differences in treatment burden and ASM regimens between the DRE and DSE groups may also have influenced the observed metabolic profiles. In addition, the potential effects of participants’ dietary habits and environmental exposures on the metabolomic profiles could not be completely excluded. Second, the sample size was relatively small, and the study had a cross-sectional design. Furthermore, although bootstrap-based internal validation was performed, the reported model performance may remain optimistic. Therefore, before any clinical application is considered, the classification performance of the models requires further validation in larger, independent, multicenter cohorts. Furthermore, causal relationships cannot be inferred from the observed associations, and the specific biological functions of the candidate metabolites also remain to be elucidated through functional studies in cellular and animal models. Third, the transcriptomic data integrated in this study were derived from brain tissue, whereas the metabolomic data were obtained from peripheral plasma. This tissue heterogeneity limits the direct biological interpretation of the pathway-level convergence between the two omics datasets.

## 5 Conclusion

In summary, this study characterized plasma metabolic alterations associated with epilepsy and anti-seizure medication responsiveness and identified three key candidate metabolites distinguishing PWE from HCs and six exploratory metabolites distinguishing DRE from DSE. These findings provide preliminary metabolic evidence for the heterogeneity of epilepsy and warrant further validation in larger independent cohorts.

## Data Availability

Publicly available datasets were analyzed in this study. The datasets are available in the NCBI Gene Expression Omnibus (GEO) repository under accession number GSE256068.

## References

[B1] Akiyama T. Saigusa D. Hyodo Y. Umeda K. Saijo R. Koshiba S. (2020). Metabolic profiling of the cerebrospinal fluid in pediatric epilepsy. Acta Med. Okayama 74 (1), 65–72. 10.18926/AMO/57955 32099251

[B2] Ajith A. Mondal S. Chattopadhyay S. Kumar A. Sthanikam Y. Chacko A. G. (2021). Mass spectrometry imaging deciphers dysregulated lipid metabolism in the human hippocampus affected by temporal lobe epilepsy. ACS Chem. Neurosci. 12 (21), 4187–4194. 10.1021/acschemneuro.1c00587 34657435

[B3] Aziz-Zadeh L. Ringold S. M. Jayashankar A. Kilroy E. Butera C. Jacobs J. P. (2025). Relationships between brain activity, tryptophan-related gut metabolites, and autism symptomatology. Nat. Commun. 16 (1), 3465. 10.1038/s41467-025-58459-1 40229237 PMC11997199

[B4] Bartova E. Stixova L. Svobodova Kovarikova A. (2025). N4-acetylcytidine and other RNA modifications in epitranscriptome: insight into DNA repair and cancer development. Epigenomics 17 (6), 411–422. 10.1080/17501911.2025.2473308 40040517 PMC11980489

[B5] Boguszewicz L. Jamroz E. Ciszek M. Emich-Widera E. Kijonka M. Banasik T. (2019). NMR-based metabolomics in pediatric drug resistant epilepsy - preliminary results. Sci. Rep. 9 (1), 15035. 10.1038/s41598-019-51337-z 31636291 PMC6803684

[B6] Bozzi Y. Borrelli E. (2013). The role of dopamine signaling in epileptogenesis. Front. Cell Neurosci. 7, 157. 10.3389/fncel.2013.00157 24062645 PMC3774988

[B7] Chen S. J. Chen C. C. Liao H. Y. Wu Y. W. Liou J. M. Wu M. S. (2022). Alteration of gut microbial metabolites in the systemic circulation of patients with parkinson's Disease. J. Park. Dis. 12 (4), 1219–1230. 10.3233/JPD-223179 35342048

[B8] Choi H. Detyniecki K. Bazil C. Thornton S. Crosta P. Tolba H. (2020). Development and validation of a predictive model of drug-resistant genetic generalized epilepsy. Neurology 95 (15), e2150–e2160. 10.1212/WNL.0000000000010597 32759205 PMC7713754

[B9] Detour J. Bund C. Behr C. Cebula H. Cicek E. A. Valenti-Hirsch M. P. (2018). Metabolomic characterization of human hippocampus from drug-resistant epilepsy with mesial temporal seizure. Epilepsia 59 (3), 607–616. 10.1111/epi.14000 29341105

[B10] Eswaran S. Mascarenhas R. Kabekkodu S. P. (2025). The ester derivative Palmitoylcarnitine abrogates cervical cancer cell survival by enhancing lipotoxicity and mitochondrial dysfunction. Cell Commun. Signal 23 (1), 213. 10.1186/s12964-025-02218-8 40319292 PMC12048960

[B11] Fisher R. S. Acevedo C. Arzimanoglou A. Bogacz A. Cross J. H. Elger C. E. (2014). ILAE official report: a practical clinical definition of epilepsy. Epilepsia 55 (4), 475–482. 10.1111/epi.12550 24730690

[B12] Gautam V. Rawat K. Sandhu A. Kumari P. Singh N. Saha L. (2021). An insight into crosstalk among multiple signaling pathways contributing to epileptogenesis. Eur. J. Pharmacol. 910, 174469. 10.1016/j.ejphar.2021.174469 34478688

[B13] Guo H. L. Wang W. J. Dong N. Zhao Y. T. Dai H. R. Hu Y. H. (2023). Integrating metabolomics and lipidomics revealed a decrease in plasma fatty acids but an increase in triglycerides in children with drug-refractory epilepsy. Epilepsia Open 8 (2), 466–478. 10.1002/epi4.12712 36808532 PMC10235554

[B14] Huang X. X. Li L. Jiang R. H. Yu J. B. Sun Y. Q. Shan J. (2024). Lipidomic analysis identifies long-chain acylcarnitine as a target for ischemic stroke. J. Adv. Res. 61, 133–149. 10.1016/j.jare.2023.08.007 37572732 PMC11258661

[B15] Hyppönen J. Paanila V. Äikiä M. Koskenkorva P. Könönen M. Vanninen R. (2023). Progressive myoclonic epilepsy type 1 (EPM1) patients present with abnormal 1H MRS brain metabolic profiles associated with cognitive function. Neuroimage Clin. 39, 103459. 10.1016/j.nicl.2023.103459 37541097 PMC10412857

[B16] Ibáñez C. Simó C. Barupal D. K. Fiehn O. Kivipelto M. Cedazo-Mínguez A. (2013). A new metabolomic workflow for early detection of alzheimer's disease. J. Chromatogr. A 1302, 65–71. 10.1016/j.chroma.2013.06.005 23827464

[B17] Jiang X. W. Lu H. Y. Xu Z. Liu T. Y. Wu Q. Yang Y. (2018). *In silico* analyses for key genes and molecular genetic mechanism in epilepsy and alzheimer's disease. CNS Neurol. Disord. Drug Targets 17 (8), 608–617. 10.2174/1871527317666180724150839 30047339

[B18] Kang J. H. Toita R. Kawano T. Murata M. Kano A. (2025a). Phospholipids and their metabolites as diagnostic biomarkers of human diseases. Prog. Lipid Res. 99, 101340. 10.1016/j.plipres.2025.101340 40409729

[B19] Kang W. Han Q. Chen M. Xue L. Zhao Z. Liu R. (2025b). Alterations in serum metabolomics predict drug-resistant epilepsy. Sci. Rep. 16 (1), 1003. 10.1038/s41598-025-30741-8 41339472 PMC12783295

[B20] Kaur M. Porel P. Patel R. Aran K. R. (2025). Kynurenine pathway in epilepsy: unraveling its role in glutamate excitotoxicity, GABAergic dysregulation, neuroinflammation, and mitochondrial dysfunction. Neurotox. Res. 43 (2), 18. 10.1007/s12640-025-00738-2 40153181

[B21] Kuhn M. Sühs K. W. Akmatov M. K. Klawonn F. Wang J. Skripuletz T. (2018). Mass-spectrometric profiling of cerebrospinal fluid reveals metabolite biomarkers for CNS involvement in varicella zoster virus reactivation. J. Neuroinflammation 15 (1), 20. 10.1186/s12974-017-1041-0 29343258 PMC5773076

[B22] Kwan P. Arzimanoglou A. Berg A. T. Brodie M. J. Allen Hauser W. Mathern G. (2010). Definition of drug resistant epilepsy: consensus proposal by the ad hoc task force of the ILAE commission on therapeutic strategies. Epilepsia 51 (6), 1069–1077. 10.1111/j.1528-1167.2009.02397.x 19889013

[B23] Lai W. Du D. Chen L. (2022). Metabolomics provides novel insights into epilepsy diagnosis and treatment: a review. Neurochem. Res. 47 (4), 844–859. 10.1007/s11064-021-03510-y 35067830

[B24] Levi I. Gurevich M. Perlman G. Magalashvili D. Menascu S. Bar N. (2021). Potential role of indolelactate and butyrate in multiple sclerosis revealed by integrated microbiome-metabolome analysis. Cell Rep. Med. 2 (4), 100246. 10.1016/j.xcrm.2021.100246 33948576 PMC8080254

[B25] Lian X. Liu Z. Liu S. Jin L. Wu T. Chen Y. (2024). Alterations in serum metabolomics during the first seizure and after effective control of epilepsy. Sci. Rep. 14 (1), 19180. 10.1038/s41598-024-68966-8 39160238 PMC11333619

[B26] Liang Z. Zheng Z. Guo Q. Tian M. Yang J. Liu X. (2023). The role of HIF-1alpha/HO-1 pathway in hippocampal neuronal ferroptosis in epilepsy. iScience 26 (11), 108098. 10.1016/j.isci.2023.108098 37876811 PMC10590818

[B27] Mondal S. Nandy A. Dande G. Prabhu K. Valmiki R. R. Koner D. (2024). Mass spectrometric imaging of anionic phospholipids desorbed from human hippocampal sections: discrimination between temporal and nontemporal lobe epilepsies. ACS Chem. Neurosci. 15 (5), 983–993. 10.1021/acschemneuro.3c00693 38355427

[B28] Mujagic Z. Kasapi M. Jonkers D. M. Garcia-Perez I. Vork L. Weerts Z. (2022). Integrated fecal microbiome-metabolome signatures reflect stress and serotonin metabolism in irritable bowel syndrome. Gut Microbes 14 (1), 2063016. 10.1080/19490976.2022.2063016 35446234 PMC9037519

[B29] Tan Q. Sun H. Wang W. Wu X. Hao N. Su X. (2018). Quantitative MR spectroscopy reveals metabolic changes in the dorsolateral prefrontal cortex of patients with temporal lobe epilepsy. Eur. Radiol. 28 (11), 4496–4503. 10.1007/s00330-018-5443-x 29728816

[B30] Thijs R. D. Surges R. O'Brien T. J. Sander J. W. (2019). Epilepsy in adults. Lancet 393 (10172), 689–701. 10.1016/S0140-6736(18)32596-0 30686584

[B31] Trezzi J. P. Galozzi S. Jaeger C. Barkovits K. Brockmann K. Maetzler W. (2017). Distinct metabolomic signature in cerebrospinal fluid in early Parkinson'S disease. Mov. Disord. 32 (10), 1401–1408. 10.1002/mds.27132 28843022

[B32] Wang H. Zhao Y. Zhang D. Li J. Yang K. Yang J. (2024). Neuroprotective effects of quinpirole on lithium chloride pilocarpine-induced epilepsy in rats and its underlying mechanisms. Eur. J. Med. Res. 29 (1), 121. 10.1186/s40001-024-01694-x 38355613 PMC10865707

[B33] Zarnowska I. M. (2020). Therapeutic use of the ketogenic diet in refractory epilepsy: what we know and what still needs to be learned. Nutrients 12 (9), 2616. 10.3390/nu12092616 32867258 PMC7551948

[B34] Zhang Y. Li J. Li J. Wang Y. Cui M. Li W. (2026). Cross-sectional and longitudinal associations of circulating bile acids and dementia: a systematic review. Geroscience 48 (3), 4789–4804. 10.1007/s11357-025-01790-8 40699484 PMC13356254

