# Supplementary material for: Plasma untargeted metabolomics reveals metabolic signatures associated with epilepsy and anti-seizure medication responsiveness
Source: Front Mol Biosci. 2026 Sep 23;13:1885513. doi: 10.3389/fmolb.2026.1885513 (PMC13642817; doi:10.3389/fmolb.2026.1885513)
Supplement: Supplementary file 1 [file DataSheet1.pdf]

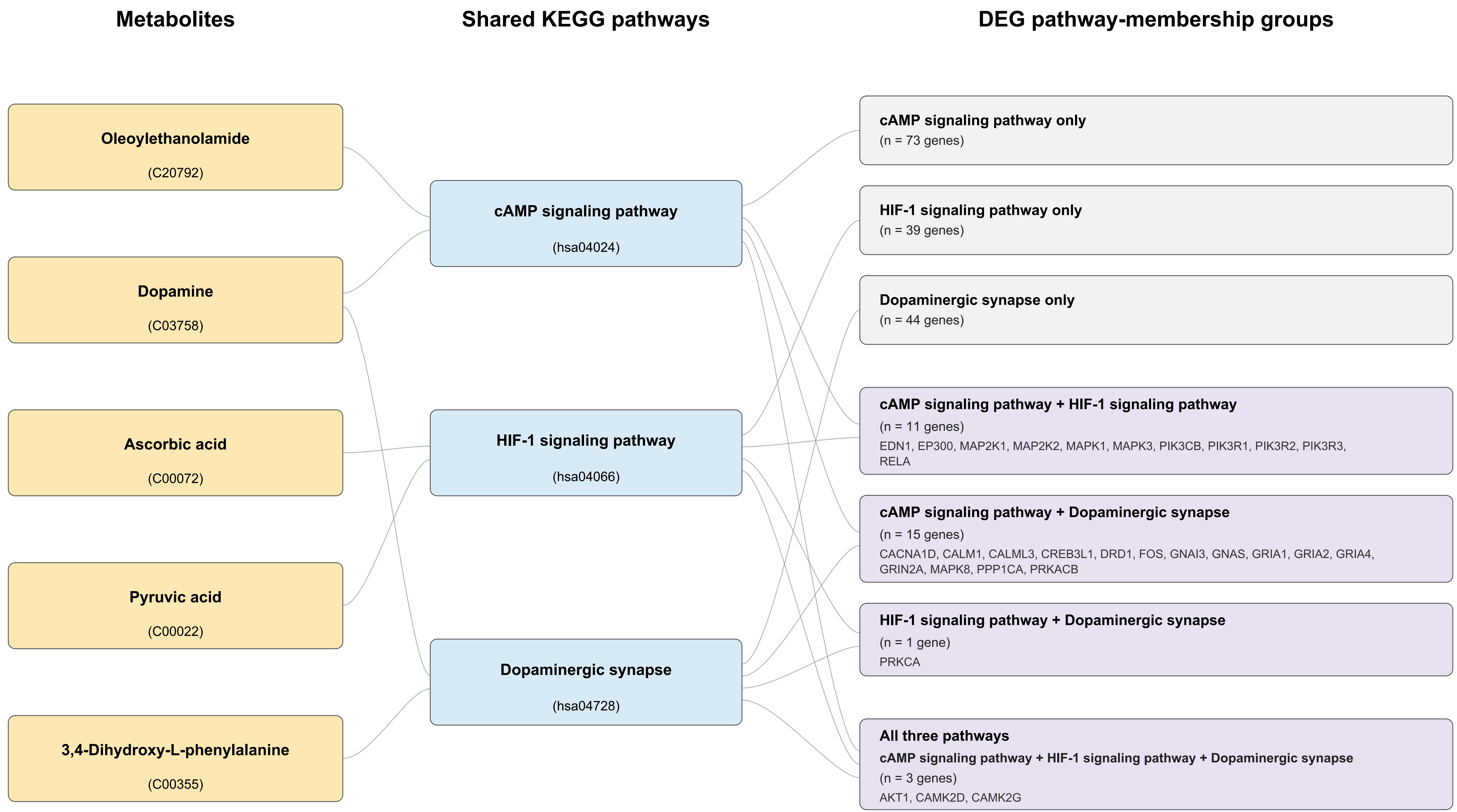
Metabolites
Shared KEGG pathways
DEG pathway-membership groups
Oleoylethanolamide
(C20792)
Dopamine
(C03758)
Ascorbic acid
(C00072)
Pyruvic acid
(C00022)
3,4-Dihydroxy-L-phenylalanine
(C00355)
cAMP signaling pathway
(hsa04024)
HIF-1 signaling pathway
(hsa04066)
Dopaminergic synapse
(hsa04728)
cAMP signaling pathway only
(n = 73 genes)
HIF-1 signaling pathway only
(n = 39 genes)
Dopaminergic synapse only
(n = 44 genes)
cAMP signaling pathway + HIF-1 signaling pathway
(n = 11 genes)
EDN1, EP300, MAP2K1, MAP2K2, MAPK1, MAPK3, PIK3CB, PIK3R1, PIK3R2, PIK3R3, RELA
cAMP signaling pathway + Dopaminergic synapse
(n = 15 genes)
CACNA1D, CALM1, CALML3, CREB3L1, DRD1, FOS, GNAI3, GNAS, GRIA1, GRIA2, GRIA4, GRIN2A, MAPK8, PPP1CA, PRKACB
HIF-1 signaling pathway + Dopaminergic synapse
(n = 1 gene)
PRKCA
All three pathways
cAMP signaling pathway + HIF-1 signaling pathway + Dopaminergic synapse
(n = 3 genes)
AKT1, CAMK2D, CAMK2G
