# Supplementary material for: Plasma untargeted metabolomics reveals metabolic signatures associated with epilepsy and anti-seizure medication responsiveness
Source: Front Mol Biosci. 2026 Sep 23;13:1885513. doi: 10.3389/fmolb.2026.1885513 (PMC13642817; doi:10.3389/fmolb.2026.1885513)
Supplement: Supplementary file 2 [file DataSheet2.pdf]

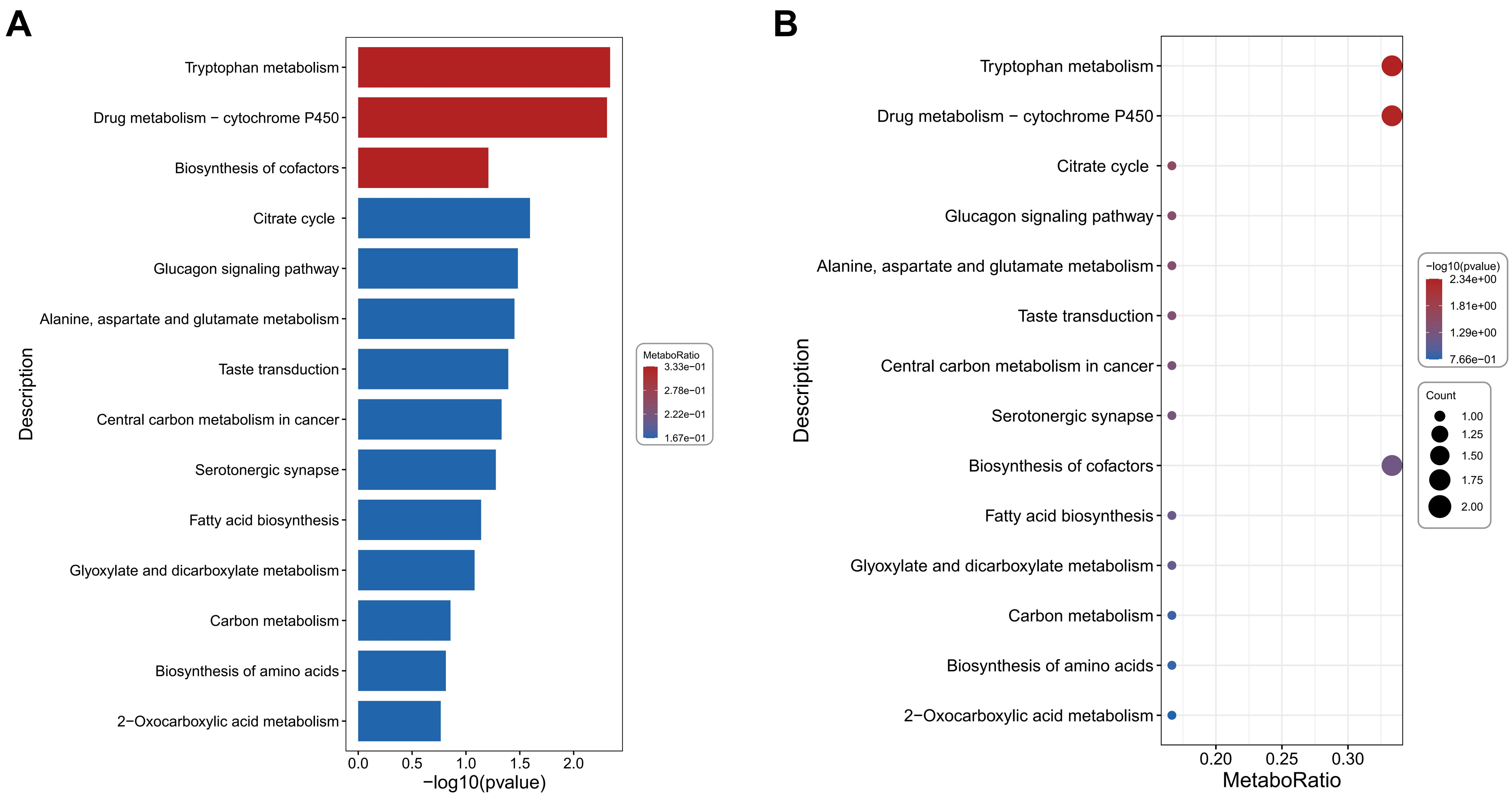

Supplementary Figure 2 KEGG enrichment analysis of differential metabolites between the DRE and DSE groups.
